# Outcomes of reconstructive techniques in breast cancer using BCCT. core software

**DOI:** 10.1186/s12957-024-03343-3

**Published:** 2024-03-23

**Authors:** Sevgi Kurt, Ahmet Serkan İlgün, Enver Özkurt, Gürsel Soybir, Gül Alço, Çağlar Ünal, Filiz Elbüken Çelebi, Tomris Duymaz, Tuğba Kayan Tapan, Naziye Ak, Çetin Ordu, Vahit Özmen

**Affiliations:** 1 Department of Plastic, Reconstructive and Aesthetic Surgery, Istanbul Florence Nightingale Hospital, Istanbul, Turkey; 2 Department of Surgery, Mater Dei Hospital, Msida, Malta; 3 Department of General Surgery, Istanbul Florence Nightingale Hospital, Istanbul, Turkey; 4 Department of General Surgery, Memorial Şişli Hospital, Istanbul, Turkey; 5 Department of Radiation Oncology, Gayrettepe Florence Nightingale Hospital, Istanbul, Turkey; 6 Division of Medical Oncology, Department of Internal Medicine, Kartal Dr. Lütfi Kırdar City Hospital, Istanbul, Turkey; 7 Department of Radiology, Yeditepe University Hospital, Istanbul, Turkey; 8 Department of Physiotherapy and Rehabilitation, Faculty of Health Sciences, Istanbul Bilgi University, Istanbul, Turkey; 9 Department of Nutrition and Dietetics, Istanbul Florence Nightingale Hospital, Istanbul, Turkey; 10 Division of Medical Oncology, Department of Internal Medicine, Istanbul Florence Nightingale Hospital, Istanbul, Turkey; 11 Division of Medical Oncology, Department of Internal Medicine, Gayrettepe Florence Nightingale Hospital, Istanbul, Turkey

**Keywords:** Breast reconstruction, Implant reconstruction, Patient outcomes, Breast cancer

## Abstract

**Background:**

Surgery remains a priority for breast cancer treatment. This study aimed to compare the cosmetic outcomes of oncoplastic patients who had undergone breast-conserving surgery, mini-LDF (latissimus dorsi flap), and immediate implant reconstruction using both the Japanese scale and the BCCT.core (The Breast Cancer Conservative Treatment cosmetic results software) program and to validate this program.

**Patients and methods:**

Patients who underwent surgery for breast cancer between 1997 and 2021 were retrospectively studied. Patients were divided into three groups: 1-those who had undergone breast-conserving surgery (245 patients, 71.3%), 2-those who had undergone mini-LDF after lumpectomy (38 patients, 11.02%), and 3- those who underwent reconstruction with implants after nipple-sparing mastectomy (61 patients, 17.68%). The patients were called for a follow-up examination, and their photos were taken. The photographs were shown to an independent breast surgeon and a plastic surgeon who was not included in the surgeries, and they were asked to evaluate and rate them according to the Japanese cosmetic evaluation scale. The same images were transferred to the computer and scored using BCCT.core.

**Results:**

The plastic and breast surgeon evaluation results showed no significant difference between the three cosmetic techniques (*p* = 0.99, 0.98).

The results of BCCT.core software measurements were similar to the results of plastic and breast surgeons (p: 0.43).

**Conclusion:**

Patients are more knowledgeable about cosmetic outcomes and expect more objective data. In this study, we used 3 different cosmetic evaluation scales. We found that these techniques give results that are compatible with each other in terms of evaluating the work done in a more concrete way. For this reason, we recommend the use of such software, which offers objective results in a subjective field such as aesthetics and is very easy to apply.

## Introduction

Breast cancer is the most common malignancy in women [1, 2]. Surgery remains a priority for treating breast cancer, and although the most commonly used method among these treatments is breast-conserving surgery, mastectomy may still be needed in 30% of patients [2–4]. The psychosocial benefits of breast reconstruction are already widely recognized [5–7], so early or late reconstruction is now recommended to patients after mastectomy. Not only breast reconstruction but also the achievement of a cosmetically pleasing outcome has a positive impact on psychosocial well-being. Therefore, it is essential to achieve a cosmetically pleasing outcome not only in reconstruction but also in breast-conserving surgery [8]. If nearly 20%-30% of breast tissue is to be removed, volume replacement techniques generally need to be used. The resulting defect is much more challenging to correct, especially after radiotherapy [9, 10], and the most commonly used volume replacement technique in our practice is the mini-latissimus dorsi flap (mini LDF).

It is important to compare the cosmetic outcomes of different surgical techniques, as this has become more important in the current time when cosmetic outcomes are of such concern. A major disadvantage of cosmetic evaluation is that it is subjective and influenced by many factors, including educational and cultural background. Numerous evaluations have been conducted to convert this subjectivity into objectivity (Harvard scale, Japanese cosmetic scale, etc [11–13]). Even with such scales, the rater's judgments may influence the outcome. The Harvard scale is a widely used method for evaluating cosmetic appearance that evaluates the overall impression using a 4-staged scale (excellent, good, fair and poor). Japanese scale scores 8 items that conducts evaluations with the highest total score of 12 points. In this study, we used the Japanese scale instead of the Harvard scale, we thought that the Japanese scale might be more objective because the evaluations are based on more factors and evaluated numerically. However these scales are still subjective and cosmetic result depends on the interpretations and experience of the assessors [14].

To eliminate this and obtain more objective data, software such as BCCT.core (The Breast Cancer Conservative Treatment cosmetic results software) was developed [11]. In this software, digital marks on nipples, axillae and sternum juguler notches identifies the breast contour with the other breast, and gives us automated measurements including breast shape, volume, deformity, nipple position, scar visibility [15].

This study aimed to compare the cosmetic outcomes of oncoplastic patients who had undergone breast-conserving surgery, mini-LDF, and immediate implant reconstruction using both the Japanese scale and the BCCT core program and to determine factors that cause poor outcomes. However, an even more important aim was to validate the BCCT. core software. BCCT.core is a program that was validated in 2007. But its intended use is more related to patients undergoing lumpectomy and breast conserving surgery. In this study, we used it to evaluate the outcomes of patients operated with different techniques.

## Patients and methods

### Patient demographics

**Fig. 1:**
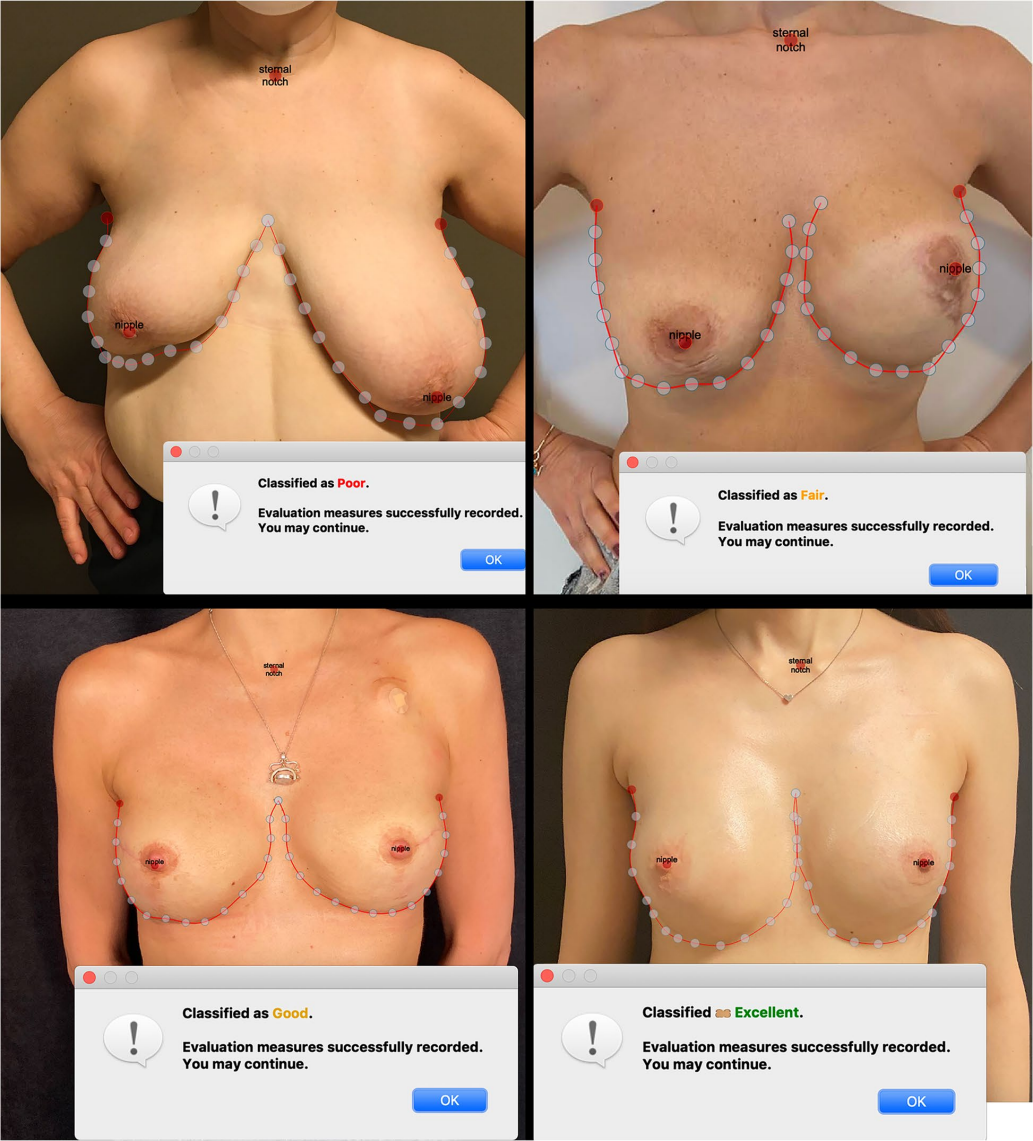
Examples of different patient scores obtained by BCCT. Core software

Patients who underwent surgery for breast cancer between 1997 and 2021 were retrospectively studied. Patients were divided into three groups: 1-those who had undergone breast-conserving surgery (BCS) (245 patients, 71.3%), 2-those who had undergone mini-LDF after breast-conserving surgery (38 patients, 11.02%), and 3- those who underwent reconstruction with implants after subcutaneous mastectomy(M + I) (61 patients, 17.68%). The same senior surgeon performed all breast-conserving surgeries, mini-LDFs, and subcutaneous mastectomies, and reconstruction with implants was performed by a total of 3 breast specified plastic surgeons. The patients were called for a follow-up examination, and their photos were taken at the same distance with the same camera (Sony Alpha A6000) and in the same light. The photographs taken were shown to an independent breast surgeon and a plastic surgeon who was not included in the surgeries, and they were asked to evaluate and rate them according to the Japanese Breast Cancer Society Cosmetic Evaluation Scale (JBCS cosmetic evaluation scale) (Table 1). The same images were transferred to the computer and scored using BCCT.core version 3.0 software. After manually placing anatomical landmarks in the program, they are automatically evaluated for asymmetry, color, and scar condition and classified into four groups: excellent, good, fair, and poor [11] (Fig. 1).

**Table 1:** Japanese cosmetic evaluation form used by plastic and general surgeons

Japanese breast cancer society cosmetic evaluation scale		
Patient Demographic properties		
**Parameter**	**Explanation**	**Score**
**Breast size** (Compare to opposite breast)	0: Significantly different 1: Slightly different 2: No difference	
**Breast shape** (Compare to opposite breast)	0: Significantly different 1: Slightly different 2: No difference	
**Incision scar**	0: Significantly visible 1: Slightly visible 2: Invisible	
**Breast firmness** (compare to the opposite breast)	0: Severe hard 1: Partial hard 2: Soft	
**Nipple areola size and shape** (compare to the opposite breast)	0: Different 1: No difference	
**Nipple areola colour** (Compare to opposite breast)	0: Different 1: No difference	
**Nipple position** (Compare to opposite breast)	0: Different 1: No difference	
**Breast sulcus** (Compare to opposite breast)	0: Difference more than 2 cm 1: Difference less than 2 cm	
	**Total score** **11–12: Excellent** **8–10: good** **5–7: fair** **0–4: poor**

### Radiotherapy

RT was applied in 37 patients (97.4%) in the mini-LDF group and in 233 patients (94.7%) in the BCS group, whereas RT was applied in 27 patients (44.3%) in the M + I group.

### Surgical technique

#### Breast-Conserving Surgery (BCS)

In patients who underwent breast-conserving surgery, inframammary sulcus or areolar incisions were used depending on the location of the tumor. The mass was excised with a solid margin, the remaining tissue was approximated to create no interstitial space, and a primary suture was performed. An incision was made separately from the axilla for sentinel lymph node biopsy.

#### Mini-LDF

After the patient was positioned, the tumor and sentinel lymph node were harvested and sent for intraoperative pathological evaluation. Then, the superior part of the latissimus dorsi muscle was found, separated from the site where it adhered to the humerus, and dissected to the scapula. After the flap was removed, a subcutaneous tunnel was prepared between the site where the tumor had been excised and the muscle, the neurovascular bundle was preserved, the tunnel was traversed, and the cavity defect created there was filled (see the related article for detailed information on surgical technique) [13].

#### Subcutaneous mastectomy + implant

Since it provides easy access to the axilla, subcutaneous mastectomy was performed through a lateral radial incision, implants were placed behind the pectoral muscle, acellular dermal matrix (Tutopatch) was used in 51 patients, an expander was placed in 10 patients, and reconstruction was performed with permanent implants in the remaining 51 patients.

### Cosmetic evaluation

Japanese Breast Cancer Society (JBCS) Cosmetic Evaluation Scale, developed by Sawai et al. [16] in 2004 and supported by the Japanese Breast Cancer Society, was used to assess cosmetic outcomes. It scores the following eight items and conducts evaluations with the highest total score of 12 points: breast size (0–2 points), breast shape (0–2 points), scar (0–2 points), breast firmness (0–2 points), nipple-areola complex (NAC) size/ shape (0–1 point), NAC color tone (0–1 point), nipple position (0–1 point), and position of the maximum descent point of the breast (0–1 point). In this method, total scores of 11 to 12, 8 to 10, 5 to 7, and 0 to 4 points were defined as "excellent," "good," "fair," and "poor." An "excellent" rating means the treated breast was nearly identical to the untreated breast. A "good" rating means that the treated breast differed slightly from the untreated breast. A "fair" rating means that the treated breast was not seriously distorted but clearly different from the untreated breast, while a "poor" rating means that the treated breast was seriously distorted. Although this scale was easy to use, it was subjective [17].

The objective evaluation was used as an objective tool for cosmesis evaluation. The software analyzed cosmetic outcomes by marking the jugular notch and both nipples and outlining the breasts with lines. The endpoints included the breast retraction assessment, lower breast contour, upward nipple retraction, breast compliance evaluation, breast contour length difference, breast area difference, and breast overlap difference. In addition, the breast image was divided into 12 fractions by 30 degrees each. Color and scar assessments were conducted simultaneously to compare the left and right breasts. The software automatically conducted all the items. Eventually, four staged evaluation results are procured as follows: "excellent," "good," "fair," and "poor." [17].

### Statistical analysis

The statistical analysis was performed using the IBM SPSS version 22 program package. The variables were examined through visual methods like histograms and probability plots and analytical methods like Kolmogorov–Smirnov/Shapiro–Wilk's test to assess whether they were normally distributed. Descriptive analyses were presented using medians for non-normally distributed parameters such as age, tumor size, follow-up time, and ordinal variables. The Chi-square test was used to compare proportions in different groups. Since JBCS Cosmetic Evaluation Scale Scores were not normally distributed, Kruskal‒Wallis tests were conducted to compare these parameters. The interrater reliability between the plastic surgeon, breast surgeon, and BCCT. core scores were determined using the intraclass correlation coefficient (ICC). To identify factors associated with poor cosmetic outcomes, the results obtained with the JBCS Cosmetic Evaluation Scale and BCCT. core software were re-grouped as poor and others. Logistic regression (enter) was used for univariate analyses to identify variables associated with poor outcomes for every parameter. For multivariate analyses, possible factors identified with univariate analyses were further entered all together into logistic regression analyses (Backward LR) to determine independent predictors of poor cosmetic outcomes. Model fit was assessed using Hosmer–Lemeshow's goodness of fit statistics. A 5% type-I error level was used to infer statistical significance.

## Results

As a result of retrospective scans, 344 patients were called, photographed, and included in the study. Of these, 38 patients underwent mini-LDF, 61 patients underwent subcutaneous mastectomy followed by implant reconstruction, and the remaining 245 patients underwent breast-conserving surgery (BCS). The medianage of the patients was 46 years (25–75), and there was no significant difference between the three groups in terms of age (p: 0.014).

The median follow-up time was 38 (6–288) months. It was calculated as a median of 62.5 (22–123) months in the mini-LDF group, 20 (8–171) months in the subcutaneous mastectomy and implant group, and 36.5 (6–288) months for BCSpatients. The follow-up time difference between the groups was significant (*p* < 0.001).

A total of 153 of the patients had tumors located in the left breast, 153 in the right breast, and 38 in both breasts. The mean follow-up duration of the patients was 53 months. In the entire patient group, 57 received neoadjuvant chemotherapy (CT), and 287 received adjuvant CT (Table 2).

**Table 2:** Patient and tumor characteristics, evaluation scores of plastic, breast surgeons and computer

		**miniLDF**	**M + I**	**BCS**	***p*-value**
**Age**	46 (25–75)	45(30–69)	45(25–61)	47(26–75)	0.014*
**Tumor size (mm)**	17(1–100)	25(2–60)	20(2–60)	15(1–100)	< 0.001*
**Tumor side**					< 0.001^#^
left	153(44.5%)	19(50%)	22(36.7%)	112(45.5%)	
right	153(44.5%)	16(42.1%)	17(28.3%)	120(48.8%)	
bilateral	38(11%)	3(7.9%)	21(35%)	14(5.7%)	
**Follow-up Time**	38(6–288)	62.5(22–123)	20(8–171)	36.5(6–288)	< 0.001
**Chemotherapy**					0.21^#^
neoadjuvant CT	57(16.6%)	4(10.5%)	14(23.3%)	39(15.9%)	
adjuvant CT	287(83.4%)	34(89.5%)	46(76.7%)	207(84.1%)	
**Radiotherapy**					< 0.001^#^
**no**	48(13.9%)	1(2.6%)	34(55.7%)	13(5.3%)	
**yes**	297(86.1%)	37(97.4%)	27(44.3%)	233(94.7%)	
**JBCS Cosmetic Evaluation Scale** **(plastic surgeon)**					
poor	21(6.1%)	3(7.9%)	5(8.3%)	13(5.3%)	
fair	136(39.7%)	16(42.1%)	23(38.3%)	97(39.6%)	
good	174(50.7%)	15(39.5%)	32(53.3%)	127(51.8%)	
excellent	12(3.5%)	4(10.5%)	0	8(3.3%)	
**JBCS Cosmetic Evaluation Scale** **(plastic surgeon)**					0.83^#^
poor/fair	157(45.8%)	19(50%)	28(46.7%)	110(45%)	
good/excellent	186(54.2%)	19(50%)	32(53.3%)	135(55%)	
**JBCS Cosmetic Evaluation Scale Score** **(plastic surgeon)**		8.5(3–12)	9(2–11)	9(3–12)	0.99*
**JBCS Cosmetic Evaluation Scale** **(breast surgeon)**					
poor	19(5.5%)	3(7.9%)	6(10%)	10(4.1%)	
fair	126(36.7%)	15(39.5%)	18(30%)	93(38%)	
good	182(53.1%)	15(39.5%)	36(60%)	131(53.5%)	
excellent	16(4.7%)	5(13.2%)	0	11(4.5%)	
**JBCS Cosmetic Evaluation Scale** **(breast surgeon)**					0.73^#^
poor/fair	145(42%)	18(47.4%)	24(39.3%)	103(41.9%)	
good/excellent	200(58%)	20(52.6%)	37(60.7%)	143(58.1%)	
**JBCS Cosmetic Evaluation Scale Score** **(breast surgeon)**		9(3–12)	9(3–11)	9(3–12)	0.98*
**BCCT.core Score**					0.43^#^
poor	22(6.4%)	2(5.3%)	5(8.3%)	15(6.1%)	
fair	110(32.1%)	15(39.5%)	13(21.7%)	82(33.5%)	
good	173(50.4%)	15(39.5%)	35(58.3%)	123(50.2%)	
excellent	38(11.1%)	6(15.8%)	7(11.7%)	25(10.2%)	
**BCCT.core Score**					0.24^#^
poor/fair	132(38.3%)	17(44.7%)	18(29.5%)	97(39.4%)	
good/excellent	213(61.7%)	21(55.3%)	43(70.5%)	149(60.6%)	

*M* + *I* Mastectomy and Implant, *LDF* Latissimus Dorsi Flap, *BCS* Breast-Conserving Surgery

^#^chi-square test

^*^Kruskal Wallis test

### Cosmetic evaluation results by plastic surgeon and brest surgeon

According to the plastic surgeon, the JBCS Cosmetic Evaluation Scale mean score was 8.5 in the mini-LDF group, 9 in the M + I group, and 9 in the BCS group. There was no significant difference in cosmesis between the three groups (*p* = 0.99) (Table 2).

As a result of the evaluation by the breast surgeon, the mean score of all groups was 9, and no cosmetic difference was observed between groups (p: 0.98) (Table 2).

When patients' JBCS Cosmetic Evaluation Scale scores were classified as "excellent," "good," "fair," and "poor," there were no significant results between groups either in the plastic surgeon or the breast surgeon evaluation. Furthermore, when poor and fair patients were re-grouped into one group and good and excellent patients were the other, there was still no difference in the cosmetic evaluation of both the plastic surgeon and the breast surgeon (Table 2).

### Cosmetic evaluation results by BCCT. core

Since there was no numerical scoring option in the BCCT. core analysis as in the Japanese scale, only poor/moderate/good/excellent ratings could be made. Among all patients, 38 (11.1%) were evaluated as cosmetically excellent, 173 (50.4%) as good, 110 (32.1%) as fair, and 22 (6.4%) as poor. BCCT. core software cosmetic evaluation between surgical groups was insignificant (*p* = 0.43). As in the JBCS Cosmetic Evaluation Scale by the plastic surgeon and breast surgeon, when patients were re-grouped as poor/fair and good/excellent, there was no significant difference in BCCT. core software cosmetic outcome (Table 2).

We also investigated the factors related to poor cosmetic results. The results obtained by the plastic surgeon and breast surgeon with the JBCS Cosmetic Evaluation Scale and BCCT. core software were re-grouped as poor and others. The univariate analyses showed that poor outcome was significantly associated with follow-up time and larger tumors, according to the plastic surgeon's perspective. However, according to the breast surgeon's perspective, poor outcome was associated with longer follow-up time, larger tumor size, tumor localization, and a higher number of retrieved lymph nodes in axillary surgery. In multivariate analyses, tumor size and follow-up time were significantly associated with the plastic surgeon's perspective. From the perspective of the breast surgeon, only longer follow-up time and tumor localization in the breast were significant (Tables 3 and 4). From the standpoint of the BCCT. core software, longer follow-up time was the only factor significantly associated with poor outcomes in both univariate and multivariate analyses (Table 5).

**Table 3:** Risk factors for poor outcome by plastic surgeon’s JBCS Cosmetic Evaluation Scale

	**Univariate analyses**			**Multivariate analyses**		
	HR	95% CI	*p*-value	HR	95% CI	*p*-value
**Age**	1.001	0.96–1.04	0.96			
**Follow-up time**	1.01	1.003–1.02	**0.006**	1.013	1.005–1.022	**0.002**
**Tumor size**	1.03	1.005–1.067	**0.021**	1.04	1.009–1.073	**0.011**
**Retrieved lymph Node number**	1.04	0.99–1.08	0.061			
**Tumor localization** (UOQ vs others)	2.56	0.96–6.88	0.061			
**Radiation therapy**	0.97	0.27–3.41	0.95			
**Surgical Procedure**						
BCS. vs mini LDF BCS vs M + I	1.53 1.6	0.41–5.66 0.54–4.67	0.52 0.39			

*BCS* Breast Conserving Surgery, *M* + *I* Mastectomy and Implant, *UOQ* Upper outer quadrant, *JBSC* Japanese Breast Conserving Surgery Cosmetic Evaluation Scale

**Table 4:** Risk factors for poor outcome by the breast surgeon’s JBCS Cosmetic Evaluation Scale

	**Univariate analyses**			**Multivariate analyses**		
	HR	95% CI	*p*-value	HR	95% CI	*p*-value
**Age**	1.01	0.97–1.05	0.62			
**Follow-up time**	1.012	1.004–1.021	**0.003**	1.02	1.01–1.03	**< 0.001**
**Tumor size**	1.036	1.004–1.068	**0.027**	1.02	0.98–1.06	0.24
**Retrieved lymph node number**	1.044	1.002–1.087	**0.041**	1.04	0.99–1.08	0.94
**Tumor localization** (UOQ vs others)	2.88	1.0–8.3	0.05	4.2	1.3–13.5	**0.016**
**Radiation therapy**	0.57	0.18–1.81	0.34			
**Surgical Procedure**						
** BCS. vs mini LDF** BCS vs M + I	2.01 2.61	0.52–7.68 0.91–7.49	0.30 0.074			

*BCS* Breast Conserving Surgery, *M* + *I* Mastectomy and Implant, *UOQ* Upper outer quadrant, *JBSC* Japanese Breast Conserving Surgery Cosmetic Evaluation Scale

**Table 5:** Risk factors for poor outcome by BCCT. core software

	**Univariate analyses**			**Multivariate analyses**		
	HR	95% CI	*p*-value	HR	95% CI	*p*-value
**Age**	1.007	0.97–1.04	0.71			
**Follow-up time**	1.009	1.001–1.017	**0.031**			
**Tumor size**	1.023	0.99–1.055	0.15			
**Retrieved lymph Node number**	1.02	0.98–1.06	0.31			
**Tumor localization** (UOQ vs others)	2.20	0.86–5.63	0.099			
**Radiation therapy**	1.63	0.36–7.21	0.52			
**Surgical Procedure**						
** BCS. vs mini LDF** BCS vs M + I	0.85 1.4	0.18–3.88 0.48–3.99	0.83 0.53			

*BCS* Breast Conserving Surgery, *M* + *I* Mastectomy and Implant, *UOQ* Upper outer quadrant

### Intraclass correlation coefficient

The interclass coefficient (ICC) analysis was done to determine the reliability of the plastic surgeon, breast surgeon, and BCCT. core score. The results showed excellent reliability with an ICC value of 0.938 (95% CI:0.923–0.95). When the patients were categorized based on the type of surgery, the ICC was found to be the lowest in patients who had undergone M + I. However, the correlation between the plastic surgeon and the breast surgeon was high in patients who underwent subcutaneous M + I, the correlation between the plastic surgeon and BCCT. core software and breast surgeon and BCCT. core software was relatively lower (*R* = 0.54, *R* = 0.63 respectively) (Tables 6 and 7).

**Table 6:** Intraclass correlation coefficient results comparing plastic surgeons, breast surgeons and BCCT core scores in different surgery types

	Intraclass Correlation	95% Confidence Interval	
		Lower Bound	Upper Bound
Single Measures	0.835	0.8	0.864
Average Measures	0.938	0.923	0.95

A two-way mixed model was used

**Table 7:** Intraclass correlation coefficient results comparing plastic surgeons, breast surgeons and BCCT core scores in different surgery types

Surgery Type		Intraclass Correlation	95% Confidence Interval	
			Lower Bound	Upper Bound
mini LDF	Single Measures	0.934	0.888	0.963
	Average Measures	0.977	0.96	0.987
mastectomy + implant	Single Measures	0.666	0.529	0.775
	Average Measures	0.857	0.771	0.912
lumpectomy	Single Measures	0.861	0.828	0.888
	Average Measures	0.949	0.935	0.96

## Discussion

Because modern treatments have extended the lifespan of patients with breast cancer, cosmetic appearance has become increasingly important [16]. Therefore, it is very important to decide which technique is more appropriate for oncoplastic patients. The size of the tumor, the presence of skin invasion, multicentricity, and breast size are very important in determining which reconstruction technique to use in these patients, so a multidisciplinary approach is of great importance. Adjuvant or neoadjuvant treatments and radiotherapy status are also very effective. For example, in a patient receiving neo-adjuvant CT, wound healing becomes much more important if radiotherapy is also required. Because it is not desirable to delay radiotherapy in any way and it is desirable to catch up with this treatment. Therefore, time is more limited, either a tissue expander may be preferred or a smaller size implant may be used. Many factors affecting the cosmetic outcome have been identified, but in most of these publications, the cosmetic assessment was subjective, whereas objective parameters were used in only a few publications [16]. In the study by Tosol Yu [18], BCCT.core was used for cosmetic assessment, and the only factor affecting the cosmetic outcome in multivariate analysis was the RT dose. In our study, the effect of RT on cosmetic appearance was not significant. Radiation therapy can now be influenced by more modern techniques and radiation oncologists experienced in breast surgery. In the study by Pezner, it was emphasized that the cosmetic outcome was worse in cases where large tumors were removed [14, 15]. However, in this study, the results of the patients who underwent breast-conserving surgery were evaluated. In our study, all three techniques were evaluated using three different methods. As a result of the BCCT score, it was found that tumor size was insignificant. In the evaluation made by plastic and breast surgeons, tumor size was found to have a significant effect on the cosmetic outcome. With neoadjuvant chemotherapy, the impact of residual tumor size on the cosmetic outcome can be reduced by shrinking the tumor or achieving a complete response. Other studies have also found that advanced age negatively affects cosmetic outcomes [14, 15], and in our study, no effect of advanced age was found according to the results of all three evaluations. In the evaluation made with all three techniques, the most important factor affecting the cosmetic outcome was the follow-up duration, and as the duration of follow-up increased, the cosmetic appearance worsened. In contrast, in the evaluation made by the breast surgeon, it was found that as the number of lymph nodes removed increased, the cosmesis was greatly affected. As a result of the statistical evaluation, when all three surgical techniques were compared, it was determined that there was no significant difference in cosmetic appearance.

When we look at the studies conducted to validate the BCCT core software, we find that this program is generally compared to the 4-point Harvard scale, with the cosmetic outcome being rated excellent, good, fair, and poor in the Harvard scale and the advantage of this software is providing reproducible digital assessment of overall cosmetic results [14]. In this study this software is compared with Japanese cosmetic scale. In the concordance analysis made with the ICC, a perfect agreement was observed in all three assessment techniques, and the lowest agreement was observed in the mastectomy implant group. The difference between this study and other validation studies is that the Japanese scale was used instead of the Harvard scale. The limitations of this study include its retrospective nature and the different follow-up times of the patients. Although there were different follow-up periods in the 3 groups, the factor that most influenced the poor outcome was the long follow-up period.

In conclusion, a significant difference was not found between the cosmetic outcomes of patients who underwent breast-conserving surgery, partial mastectomy, and mini-LDF as part of treatment for breast cancer and those who underwent a subcutaneous mastectomy. In the concordance analysis made with the ICC, a perfect agreement was observed in all three assessment techniques. Nowadays patients are more knowledgeable about cosmetic outcomes and expect more objective data. In this study, we used 3 different cosmetic evaluation scales. We found that these techniques give results that are compatible with each other in terms of evaluating the work done in a more concrete way. For this reason, we recommend the use of such software, which offers objective results in a subjective field such as aesthetics and is very easy to apply.

## Data Availability

All patients were from our breast center archive for more than 30 years, and all the information was retrieved from this specific breast cancer archive. If required, we can share our data with the journal editorial board or reviewers.

## Abbreviations

BCS: Breast-conserving surgery
M + I: Subcutaneous mastectomy and implant reconstruction
LDF: Latissimus dorsi flap
BCCT. core: The Breast Cancer Conservative Treatment cosmetic results software)
RT: Radiotherapy
ICC: Intraclass correlation coefficient
CT: Chemotherapy
